# Osteology of the Feeding Apparatus of Chilean Flamingo *Phoenicopterus chilensis* (Aves: Phoenicopteridae)

**DOI:** 10.1002/jmor.70116

**Published:** 2026-03-05

**Authors:** Oscar Aldana Ardila, Caio J. Carlos

**Affiliations:** ^1^ Laboratório de Comportamento, Ecologia e Filogenia de Aves Aquáticas, CEFALAB. Programa de Pós‐graduação em Biologia Animal, Departamento de Zoologia Universidade Federal do Rio Grande do Sul Porto Alegre Brazil

**Keywords:** feeding behavior, functional anatomy, osteology, skull morphology

## Abstract

Flamingos (Phoenicopteridae) feed using a uniquely adapted bill that extracts small particles from the water and sediments. This study provides a detailed osteological description of the skull of the Chilean Flamingo (*Phoenicopterus chilensis*), with particular emphasis on feeding‐related features. The skull exhibits a broad, flattened frontal region that articulates with an elongated upper jaw, which is deflected ventrally at approximately 45° and aligns seamlessly with the laterorostrally curved mandible, forming a configuration well suited for filter feeding. The mandible exhibits a low mechanical advantage, indicating an adaptation for rapid and repetitive movements rather than forceful biting. Jaw muscle attachment sites, including the fossa subtemporalis, fossa temporalis, and fossa ventralis palatini, are reduced, suggesting the presence of relatively small muscles that favor speed and precision.

## Graphical abstract

1. The skull features a broad, dorsoventrally flattened frontal region articulating with an elongated upper jaw that is complemented by the mandible's downward bend along its proximal half.

2. The low mechanical advantage in the mandible is attributed to the reduced and shallow insertion areas of the adductor muscles, which are positioned close to the jaw joint.

3. The tubular cavity formed between the *rami* provides suitably space for tongue movements, enhancing the efficiency of the water‐pumping system during filter feeding.

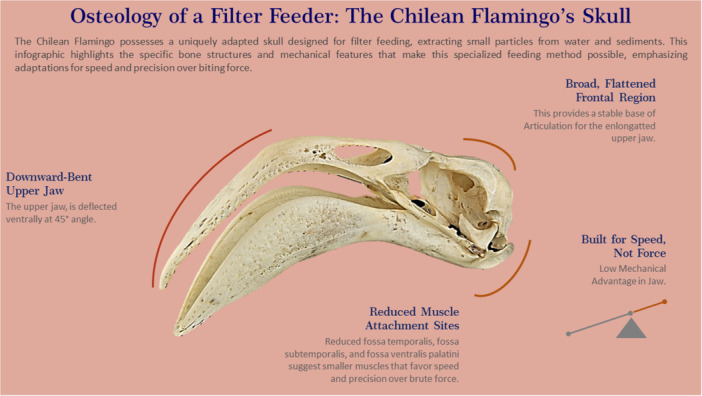

## 1 Introduction

Flamingos are large, long‐legged wading birds with pink or reddish plumage, inhabiting tropical and subtropical lakes, mudflats, and shallow lagoons across the Americas, Africa, Asia, and Europe (Winkler et al. 2020). Presently, these birds are classified into a single family, Phoenicopteridae, with three genera and six valid species, namely, the American Flamingo *Phoenicopterus ruber* Linnaeus, 1758, the Greater Flamingo *Phoenicopterus roseus* Pallas, 1811, the Chilean Flamingo *Phoenicopterus chilensis* Molina, 1782, the Lesser Flamingo *Phoeniconaias minor* (É. Geoffroy Saint‐Hilaire, 1798), the Andean Flamingo *Phoenicoparrus andinus* (Philippi, 1854), and the James Flamingo *Phoenicoparrus jamesi* (Sclater, 1886) (Winkler et al. 2020; Gill et al. 2023).

The flamingos' inter‐ and intragroup phylogenetic relationships have been the subject of an ongoing debate, shaped by both anatomy/morphology and molecular genetics. Historically, flamingos were often classified within the Ciconiiformes, alongside storks (Ciconiidae), herons (Ardeidae), ibises and spoonbills (Threskiornithidae), with the last‐named group proposed as their closest relative (Olson and Feduccia 1980; Cracraft 1981; Sibley et al. 1990). However, over the last two decades, both morphological and genetic evidence have increasingly supported a sister‐group relationship between flamingos and grebes (Podicipedidae) (e.g., Tuinenf et al. 2001; Cracraft et al. 2004; Chubb 2004; Mayr 2004; Ericson et al. 2006; Manegold 2006; Hackett et al. 2008; Morgan‐Richards et al. 2008; Jarvis et al. 2014; Prum et al. 2015), and this clade has been named Mirandornithes (Sangster 2005). Moreover, studies employing both mitochondrial and nuclear markers supported a split of the six flamingo species into two more inclusive clades (Torres et al. 2014; Frias‐Soler et al. 2022), congruent with an earlier study that found that flamingos can be separated into two groups based on their bill structure (Jenkin 1957).

Flamingos typically forage at the sediment‐water interface, where they submerge their entire head while moving steadily forward in a near‐straight path. In certain habitats, such as shallow lakes with sparse *Ruppia* beds that harbor invertebrates, these birds employ different foraging behaviors to disturb the sediment. Occasionally, these birds can filter planktonic cladocerans from the water surface while swimming (Delfino and Carlos 2021; del Hoyo et al. 2024).

Flamingos are among the few birds, along with certain ducks and swans (Anatidae) and prions (Procellariidae) that have evolved a specialized filter‐feeding mechanism (Klages and Cooper 1992). The flamingo feeding mechanism relies on its large piston‐like tongue and comb‐like lamellae of epidermal origin, which enable it to pump and filter water to capture small brine shrimp, copepods, sediments, and other food particles (Jenkin 1957; Crome 1985; Klages and Cooper 1992; Brent Gurd 2006; Kouzov et al. 2021). Within flamingos, two distinct feeding morphotypes are recognized: the ‘shallow‐keeled’ and ‘deep‐keeled’ bills (Jenkin 1957). The American, Greater, and Chilean Flamingos possess shallow‐keeled bills, characterized by an upper jaw that is as wide as the lower and broader lamellae, allowing the filtration of larger food items, such as insect larvae and crustaceans. In contrast, the Lesser, Andean, and James Flamingos exhibit a deep‐keeled bill, where the upper jaw is narrower than the lower, and finer lamellae allowing the filtration of microalgae and diatoms (Jenkin 1957; Mascitti and Kravetz 2002; Zweers et al. 1995).

Shufeldt (1901) provided the first complete osteological description of the American flamingo. He examined each skeletal element in detail and compared its morphology with that of ibises, ducks, and swans to identify relevant similarities and differences, thereby clarifying its taxonomic affinities. The feeding apparatus of flamingos was later studied comprehensively by Jenkin (1957), who described the structure, size, and variation of the bill, including the lamellae and tongue, as well as the sensory organs involved in the filter‐feeding process. In addition, she offered a detailed account of the filtration mechanism and the bill structures involved, featuring interspecific variation across species. More recently, Mascitti and Kravetz (2002) conducted a comparative study focused on the three South American flamingo species. The authors analyzed the bill shape, highlighting how morphological differences in bill structure are adaptations to specific ecological niches, enabling each species to effectively exploit different food resources.

Zweers et al. (1995) investigated the filter‐feeding mechanism in the American and Greater Flamingos by describing the oropharyngeal integument, including the tongue, taste buds, lamellae, and related structures. Through anatomical dissections, high‐speed videography, and biomechanical modeling, the authors analyzed the filtration system and demonstrated how the tongue, lamellae, and bill movements are coordinated during feeding. Ortega‐Jimenez et al. (2025) demonstrated that Chilean Flamingos employ water vortices to enhance prey capture. Furthermore, the authors working with captivity‐trained flamingos and 3D‐printed anatomical models of the head and the tarsometatarsus. They discovered that synchronized motions of the head, bill, and foot can generate vortical traps allowing to concentrate small prey such as brine shrimp, thereby improving feeding efficiency even in turbid conditions.

Rooth (1965) provided a detailed account of the habitat, diet, and reproduction of the American Flamingo on Bonaire. The author also focuses his study on the mechanics of the filter‐feeding apparatus, particularly the tongue, and its function as a piston‐like water pump within the oral cavity. He demonstrated how the integration of tongue hydraulics and bill kinematics underlies the flamingo's ability to exploit benthic resources in hyper‐saline lagoons such as those of the Netherlands Antilles.

The Chilean Flamingo is a large wading bird (105 cm in length; 1720–2500 g) closely related to the American and Greater Flamingo (Torres et al. 2014; Frias‐Soler et al. 2022; del Hoyo et al. 2024). Chilean Flamingos primarily consume aquatic invertebrates, including copepods (Centropagidae), cladocerans (Daphniidae), ostracods, amphipods, and polychaete worms (Nereididae) (Tobar et al. 2014; Aldana‐Ardila and Carlos 2021). They also prey on the larvae and pupae of lake flies (Chironomidae) and brine flies (Ephydridae) (Gallardo and Rodriguez 1992; Polla et al. 2018; Aldana‐Ardila and Carlos 2021; del Hoyo et al. 2024).

In this study, we aimed to expand the current understanding of the feeding mechanisms in flamingos by providing a detailed osteological description of the cranium and mandible of the Chilean Flamingo. Our analysis focuses on the skeletal structures associated with the muscles involved in jaw movement (i.e., *Musculi mandibulae*, sensu Vanden Berge and Zweers 1993). Furthermore, flamingos exhibit specialized foraging behaviors, including sweeping the head upside down through shallow water, using the tongue to pump water through the bill, and filtering food via the lamellae. These birds, also employ additional strategies, such as skimming, stamping, and walking to capture prey (Jenkin 1957; Rooth 1965; Zweers et al. 1995). Accordingly, we hypothesize that their jaw morphology is optimized for rapid movements rather than forceful biting.

## 2 Materials and Methods

### 2.1 Specimens Examined and Descriptions

We examined 38 skulls of adult Chilean Flamingos (*P. chilensis*), identified by the complete closure of cranial sutures (Jollie 1957). The sample comprised 8 males, 4 females, and 26 individuals of undetermined sex. Specimens were housed in the collections of Museu de Ciências Naturais da Secretaria do Meio Ambiente e Infraestrutura de Porto Alegre, Porto Alegre, Brazil (MCN); the Natural History Museum, Tring, UK (NMHUK); the National Museum of Natural History, Smithsonian Institution, Washington, DC, United States (USNM); and the American Museum of Natural History, New York, United States (AMNH) (Supporting Information Table 1).

For anatomical nomenclature, we primarily followed the *Nomina Anatomica Avium* (Baumel and Witmer 1993; Baumel and Raikow 1993; Vanden Berge and Zweers 1993). However, for lacrimal bone (*Os lacrimale*) structures, we adopted Cracraft (1968) terminology, and for the bony palate (*palatum osseum*), we followed Zusi and Livezey (2006). Any additional deviations from the *Nomina Anatomica Avium* are explicitly indicated in the text.

**Figure 1:**
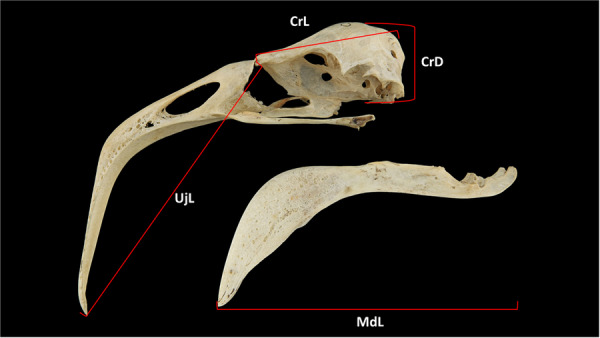
Measurements of the skull of Chilean Flamingo *Phoenicopterus chilensis*: **CrL**, cranium length; **UjL**, upper jaw length; **CrD**, cranium depth; **MdL**, Mandible length. The measurements are in millimeters mm. Not to scale.

Each skull was examined under an 8× magnifying glass and photographed using a Nikon D5500 digital camera with a 60‐mm f/2.8 Nikon macro lens. We recorded the following cranial measurements (Burger 1978): cranium length, cranium depth, as well as upper jaw (maxilla) and mandible (mandibula) length (Figure 1). All components of the hyoid apparatus (e.g., paraglossum, basihyale, urohyale, ceratobranchiale, and epibranchiale) were described based on the complete apparatus of specimen USNM 344840. In addition, dorsal and lateral views of the hyoid apparatus are illustrated.

Cranium length was measured along the longitudinal axis from the craniofacial bending zone (*zona flexoria craniofacialis*) to the posteriormost point of the occiput. Cranial depth was recorded from the center of the frontal bone (*Os frontale*) to the base of the parasphenoid lamina (*lamina parasphenoidalis*). Upper jaw length was measured along the longitudinal axis from the craniofacial hinge to the distal tip of the upper jaw (*apex rostri maxillae*), and mandible length was taken from the retroarticular process (*processus retroarticularis*) to the distal tip of the mandible (Figure 1). All measurements were obtained using digital vernier caliper to the nearest 0.1 mm (Supporting Information Table 1).

### 2.2 Morphological Data Analyses and Mechanical Advantage (MA) in Flamingos

Given the documented sexual dimorphism in Chilean Flamingos, where males are generally larger than females (Montalti et al. 2012), we performed an Independent *t*‐test to assess differences in skull measurements between sexes (Scherer et al. 2014). Furthermore, to assess normality and equality of variances in each measurement, we use a Shapiro–Wilk and a Levene's test, applying a Student's *t*‐test if variances are equal, and Welch's *t*‐test otherwise. To perform all statistical tests, we used the ‘scipy. stats’ Python package, considering a statistical significance level of α = 0.05 (Table 1).

**Table 1:** Linear discriminant analysis and Mechanical advantage of the skull of Chilean flamingo *Phoenicopterus chilensis*. All measurements are in millimeters (mm).

Measurement	Sex	Mean	Std dev	T‐statistic	*p*‐value
Cranium length	M	48.53	1.82	−1.0511	0.3058
Cranium length	F	49.39	1.7	−1.0511	0.3058
Cranium depth	M	29.41	1.21	0.9949	0.3317
Cranium depth	F	28.91	0.8	0.9949	0.3317
Upper jaw length	M	106.42	5.65	4.7528	0.0001
Upper jaw length	F	95.47	3.15	4.7528	0.0001
Mandible length	M	139.87	5.41	1.8272	0.0826
Mandible length	F	135.57	4.43	1.8272	0.0826

Due to the high number of specimens with undetermined sex, we conducted a linear discriminant analysis based on morphometric variables (Dechaume‐Moncharmont et al. 2011), to determine whether any of the unknown‐sex specimens could be assigned to either sex. To perform this analysis, we used the Python package ‘scikit‐learn’ and selected 12 specimens of indeterminate sex with complete morphometric data (Table 2). Finally, we examined morphological differences between the sexes in the 22 analysed specimens of known sex, and any differences identified were noted in the text.

**Table 2:** Independent t‐tests, and normality and equality of variances assessments in measurements of the skull of Chilean Flamingo *Phoenicopterus chilensis*.

Catalog number	Sex	Cranium length	Cranium depth	Upper jaw length	Mandible length	Predicted sex	Probability_F	Probability_M	Mechanical advantage
MCN‐164	Und	47.38	27.66	106.59	134	M	7.22E‐04	0.999278	0.122
MCN‐475	Und	51.13	29.8	110.87	137	M	1.55E‐07	1	0.125
MCN‐476	Und	50.42	NA	114.04	144	NA	—	—	0.121
MCN‐477	Und	46.81	29.21	116.51	144	M	1.11E‐08	1	—
MCN‐517	Und	50.51	29.11	111.41	137	M	2.42E‐07	1	0.124
MCN‐593	Und	50.37	32.07	115.96	146	M	2.51E‐10	1	0.125
MCN‐732	Und	47.27	30.63	113.9	139	M	1.15E‐08	1	0.124
MCN‐748	Und	45.99	30.19	106.78	132	M	2.95E‐05	0.99997	—
MCN‐750	Und	50.27	29.21	114.8	143	M	1.68E‐08	1	—
USNM 321766	Und	51.12	28.62	92.88	133	F	9.98E‐01	0.002384	—
USNM 10021	Und	47.69	27.34	98.31	131	F	8.91E‐01	0.10851	—
AMNH 30537	Und	48.12	30.32	99.88	135	M	6.71E‐02	0.932944	—
AMNH 3162	Und	51.83	29.36	91.87	140	F	1.00E + 00	0.00049	—

MA is a fundamental physical principle that quantifies the performance of a mechanical system, specifically in terms of force output relative to force input (Uicker et al. 2011). In a context of biological systems, such as mammalian jaws, MA is defined as the ratio of the length of the in‐lever (moment arm of the muscle) to the length of the out‐lever (distance from the jaw condyle to the biting point) (Morales‐García et al. 2021). In birds, MA, is defined as the ratio of the force arm to the resistance arm, and quantifies how effectively the adductor muscles can amplify force at the bite point (Zusi 1962; Raikow 1970; Burger 1978; Hildebrand et al. 2006). A higher MA indicates an adaptation for generating greater bite force, whereas a lower MA suggests a specialization for rapid jaw closure (Morales‐García et al. 2021).

**Figure 2:**
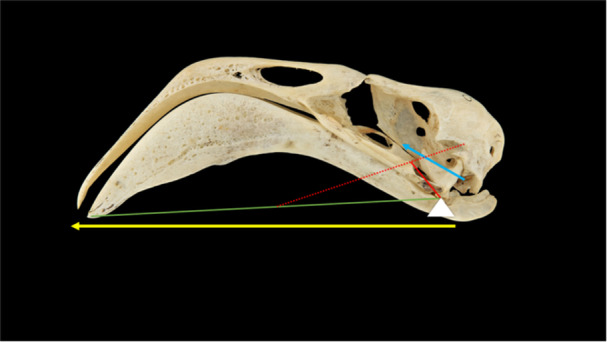
Mechanical advantage modeling of the mandible in the Chilean Flamingo *Phoenicopterus chilensis*: **yellow arrow**, resistance arm; **green line**, out lever arm length; **red dot line**, intersection point of the *fossa musculorum temporalium*; **blue arrow**, force arm; **red line**, in lever arm length.

To calculate the MA of the Chilean Flamingo's mandible, we analyzed six skulls of *P. chilensis* with a complete upper jaw, quadrate bone, and mandible (Table 2). The mandible was treated as a third‐class lever (Uicker et al. 2011), with the fulcrum positioned at the mandibular joint between the cotyles of the mandible and the condyles of the quadrate. The length of the force arm was measured from the mandibular joint to the intersection point of the fossa for muscles of the temporal region, and the resistance arm length, from the mandibular joint to the tip of the bill (Figure 2). Based on this arrangement, the following formula was applied:

$$\mathrm{MA}=\frac{\mathrm{Force}\;\mathrm{arm}\;\mathrm{length}\;(\mathrm{in}‐\mathrm{lever})}{\mathrm{Resistance}\;\mathrm{arm}\;\mathrm{length}\;(\mathrm{out}‐\mathrm{lever})}$$

## 3 Results

### 3.1 Morphological Data Analyses and Mechanical Advantage in Flamingos

The independent *t*‐test revealed a statistically significant difference between sexes only for Upper jaw length (*t* = 4.7528, *p* = 0.0001). All skull measurements exhibited equal variances between sexes, as indicated by Levene's test *p*‐values being greater than 0.05 (*p* > 0.05 for all). The Shapiro–Wilk test showed that most measurements were normally distributed for both sexes, except for Cranium depth in males, which displayed a potential deviation from normality (*p* = 0.0323) (Table 1). The linear discriminant analysis model achieved an overall classification accuracy of 92% (0.92). Its precision for predicting females was 80% (0.80), while predictions for males were correct in 100% (1.00) of cases (Table 2). MA values in the Chilean flamingo range from a minimum of 0.21 to a maximum of 0.25, a very low MA (Table 2).

### 3.2 Ossa Cranii

**Figure 3:**
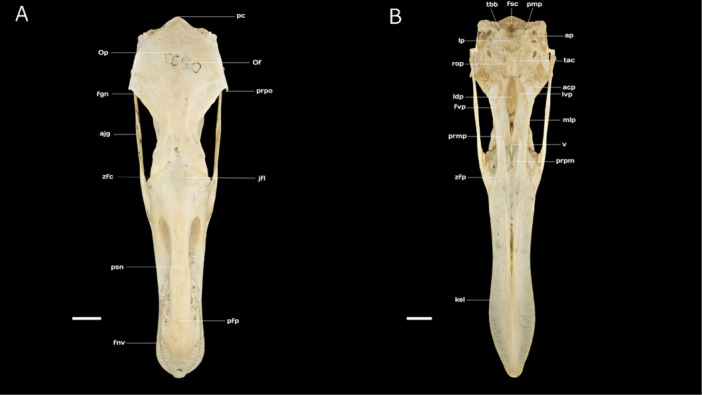
(A) Dorsal view of the skull of Chilean Flamingo *Phoenicopterus chilensis*: **pc**, prominentia cerebellaris; **Op**, Os parietale; **Of**, Os frontale; **prpo**, processus postorbitalis; **fgn**, fossa glandulae nasalis; **ajg**, arcus jugalis; **zfc**, zona flexoria craniofacialis; **jfl**, junctura frontolacrimalis; **psn**, pila supranasalis; **pfp**, processus frontalis premaxillaris; **fnv**, foramen neurovascularium. (B) Ventral view of the skull of Chilean Flamingo *P. chilensis*: **tbb**, tuberculum basilaris; **fsc**, fossa subcondylaris; **pmp**, processus medialis parasphenoidalis; **ap**, ala paraspheinoidalis; **lp**, lamina paraspheinoidalis; **tac**, tuba auditiva communis; **rop**, rostrum paraspheinoidalis; **acp**, angulus caudolateralis palatini; **lvp**, lamella ventralis pars choanalis palatini; **ldp**, lamella dorsalis pars choanalis palatini; **fvp**, fossa ventralis palatini; **mlp**, margo lateralis palatini; **prmp,** pars maxillaris palatini; **v**, vomer; **prpm**, processus palatinus maxillaris; **zfp**, zona flexoria palatina; **kel**, keel. Scale bar equal 10 mm.

The frontal region (*Os frontale*) is relatively flattened and constitutes a substantial portion of the skull roof (*calvaria*), covering approximately two‐thirds of the total skull (cranium) length. Rostrally, the frontal region articulates with the nasal region via the craniofacial bending zone (*zona flexoria craniofacialis*), which appears as a narrow transverse band in dorsal view. Caudally, the frontal region connects with the broad parietal region (Os parietale) and the postorbital process (*processus postorbitalis*), which extends ventrally and slightly rostrally, tapering at its end. The frontolacrimal suture (junctura [naso‐] *frontolacrimalis*) is located laterally to the craniofacial hinge (Figure 3A).

The parietal region (*Os parietale*) is considerably broader than the interorbital width of the frontal region, measuring approximately twice as wide. Laterally, it borders the squamosal region (*Os squamosum*) at the dorsal temporal crest (crista [lineae] *temporalis dorsalis*), which delineates the upper limit of the fossa for muscles of the temporal region (*fossa musculorum temporalium*, sensu Zusi and Livezey 2000). The two temporal crests converge at the parietal region but remain separated by a medial ridge with an average width of 2.12 mm (Figure 3A).

**Figure 4:**
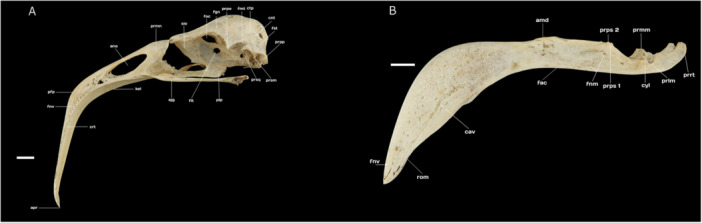
(A) Lateral view of the skull of Chilean Flamingo *Phoenicopterus chilensis*: **ctp**, crista temporalis; **fmt**, fossa musculorum temporalium; **prpo**, processus postorbitalis; **fgn**, fossa glandulae nasalis; **foc**, fonticulus orbitocranialis; **sio**, septum interorbitale; **prmn**, processus maxillaris nasalis; **ano**, apertura nasi ossea; **pfp**, processus frontalis premaxillaris; **fnv**, foramen neurovascularuim; **apr**, apex rostri; **crt**, crista tomialis; **kel**, keel; **ajg**, arcus jugalis; **fit**, fonticulus interorbitalis; **plp**, pars lateralis palatini; **prsq**, processus squamosalis; **prsm**, processus suprameaticus; **prpp**, processus paroccipitalis; **fst**, fossa subtemporalis; **cnt**, crista nuchalis transversa. (B) Lateral view of the mandible (mandibula) of Chilean Flamingo *P. chilensis*: **fnv**, foramen neurovascularuim; **rom**, rostrum mandibulae; **cav**, canaliculi neurovasculares; **fac**, fossa aditus canalis neurovascularis; **fnm**, fenestra mandibularis rostralis e fenestra mandibularis caudalis; **prps**, processus pseudocoronoidei mandibulae 1‐2; **cyl**, cotyla lateralis; **prlm**, processus lateralis mandibulae; **prrt**, processus retroarticularis; **prmm**, processus medialis mandibulae; **amd**, angulus dorsalis mandibulae. Scale bar equal 10 mm.

The squamosal region (O*s squamosum*) is bounded dorsolaterally by the dorsal temporal crest, laterocaudally by the nuchal crest (*crista nuchalis transversa*), laterorostrally by the anterior temporal crest (*crista temporalis*), and ventrolaterally by the sharply pointed suprameatic process (*processus suprameaticus*). In its dorsal portion, the fossa for muscles of the temporal region is relatively shallow compared with its parietal region. Laterally, its narrow area is bounded between the postorbital process and the squamosal process. Additionally, the squamosal region extends rostrally to form the squamosal process (*processus squamosalis*, sensu Posso and Donatelli 2006), which tapers to a point and provides the origin for the aponeurosis of the temporal muscles (*Musculus adductor mandibulae externus ventralis* and *Musculus adductor mandibulae externus rostralis lateralis*, sensu Vanden Berge and Zweers 1993). The subtemporal fossa (*fossa subtemporalis*), which accommodates the origin of the mandibular depressor muscle (*Musculus depressor mandibulae*, sensu Vanden Berge and Zweers 1993), is shallow, narrow, and short (Figure 4A).

**Figure 5:**
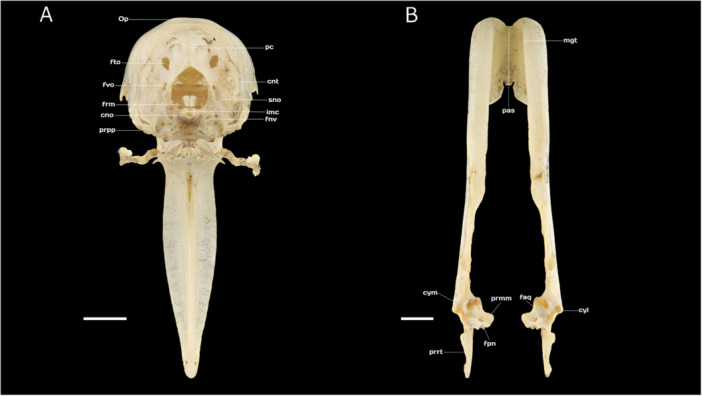
(A) Caudal view of the skull of Chilean Flamingo *Phoenicopterus chilensis*: **Op**, Os parietale; **pc**, prominentia cerebellaris; **fto**, fonticulus occipitalis; **cnt**, crista nuchalis transversa; **fvo**, foramen venae occipitalis externae; **sno**, sulcus nervi olfactorius; **frm**, foramen magnum; **imc**, incisura mediana condyli; **cno**, condylus occipitalis; **fnv**, foramen nervi vagi; **prpp**, processus paroccipitalis. (B) Dorsal view of the mandible (mandibula) of Chilean Flamingo *P. chilensis*: **pas**, pars symphisialis; **mgt**, margo tomialis; **faq**, fossa articularis quadratica; **cyl**, cotyla lateralis; **prmm**, processus medialis mandibulae; **fpn**, foramen pneumaticum; **prrt**, processus retroarticularis; **cym**, cotyla lateralis. Scale bar equal 10 mm.

The occipital region comprises the fused supraoccipital (O*s supraoccipitale*), exoccipital (O*s exoccipitale*), and basioccipital (O*s basioccipitale*) bones, which collectively constitute the pentagon‐shaped foramen magnum. The supraoccipital region extends ventrolaterally into the exoccipital area, with its medial section marked by the cerebellar prominence (*prominentia cerebellaris*), a subtle convexity distinguished by a medial ridge (*crista [linea] medialis*, sensu Livezey and Zusi 2006). Laterally, the occipital fontanelle (*fonticulus occipitalis*) is present, while dorsolaterally, a sulcus accommodates the foramina for the external occipital vein (*foramen venae occipitalis externae*). The bilobate occipital condyle (*condylus occipitalis*) is divided by a median notch (*incisura mediana condyli*, sensu Livezey and Zusi 2006). The exoccipital region is bordered medioventrally by the basioccipital and laterorostrally by the external acoustic meatus (*meatus acusticus externus*), which is connected via the paraoccipital process (*processus paraoccipitalis*), extending ventrally with a bluntly rounded tip (Figure 5A).

The basioccipital region, located on the ventral surface of the skull, forms the posterior skull base, positioned between the basisphenoid bone (O*s basisphenoidale*) anteriorly and the foramen magnum posteriorly. It is flanked laterally by the exoccipital bone and houses several important foramina, including the carotid canal (*ostium canalis carotici*), the ophthalmic nerve canal (*ostium canalis ophthalmici externi*), and the openings for the glossopharyngeal (*foramen nervi glossopharyngealis*), vagus (*foramen nervi vagi*), and hypoglossal (*canales nervi hypoglossi*) nerves. Caudolaterally, the nuchal crest (*crista nuchalis lateralis*) borders this region (Figure 5A).

The braincase floor is primarily formed by the basisphenoid (O*s basisphenoidale*) and basioccipital (O*s basioccipitale*) bones. Caudally, this region comprises two distinct portions:

1. The parasphenoid rostrum (*rostrum parasphenoidale*)—an elongated, cone‐shaped structure that forms the ventral part of the *ala parasphenoidalis* and fuses dorsally with the interorbital septum (*septum interorbitale*). It articulates rostroventrally with the pterygoid bone (O*s pterygoideum*) at the articular face of the pterygoid (*facies articularis pterygoidea*) and connects with the palatine bone (O*s palatinum*) via the articular face of the palatine (*facies articularis palatina*) (Figure 3B).
2. The parasphenoid lamina (*lamina parasphenoidalis*) a well‐expanded, wedge‐shaped structure that exhibits clearly defined caudal boundaries when viewed ventrally. In its most ventrocaudally portion and surrounding the subcondylar fossa (*fossa subcondylaris*), there are two discrete tubercula basilaria (*tuberculum basilaris*) beside the *processus paraocciptalis*. Caudally, this region articulates with the basisphenoid bone (O*s basisphenoidale*) through the *crista basilaris transversa* (Figure 3B).

On the ventral surface of the basisphenoid, posterior to the prominent projection of the parasphenoid, lies a depression known as the *tuba auditiva communis*. This depression forms a characteristic rounded wedge near the *ala parasphenoidalis* and is broad and well‐developed, extending over more than half of the *rostrum parasphenoidale* (Figure 3B).

The *septum interorbitale* is limited rostrodorsally to the cranial roof of the frontal through the orbitocranial fontanelle (*fonticulus orbitocranialis*). Lateroventrally is limited with the *rostrum parasphenoidale* through the interorbital fontanelle (*fonticulus interorbitalis*) and the *foramen nervi optici* (Figure 4A).

### 3.3 Ossa Faciei

The lacrimal bone (*Os lacrimale*) is elongated and rod‐shaped, with its head (*caput ossis lacrimalis*) articulating firmly with the frontal and nasal bones, and positioned laterally to the craniofacial joint (*zona flexoria craniofacialis*). In addition, the head of the lacrimal bone features a short and blunt supraorbital process (*processus supraorbitalis lacrimalis*). The descending process (*processus descendens ossis lacrimalis*) is relatively short and terminates in an expanded foot (*pes lacrimalis*), which articulates with the jugal arch (*arcus jugalis*).

In the Chilean Flamingo, the upper jaw (*rostrum maxilla*, sensu Livezey and Zusi 2006) constitutes approximately 70% of the total skull length and exhibits a pronounced downward curvature over its proximal half at an angle of roughly 45°. This structure results from the fusion of the premaxillary (O*s premaxillare*), maxillary (O*s maxillare*), and nasal (O*s nasale*) bones. The *pila supranasalis* is robust and thin, with a smooth surface. The upper jaw articulates ventrocaudally with the palatine bone (O*s palatinum*), dorsocaudally with the frontal bone (O*s frontale*), and laterocaudally with the jugal arch (Figure 4A).

The nasal bone (O*s nasale*) articulates laterally with the premaxillary bone through the premaxillary process (*processus premaxillaris*), contributing to the dorsal boundary of the elliptical external nasal openings (*apertura nasi [nasalis] ossea*), which are holorhinal in type and longer than they are tall. The lateral maxillary process of the nasal bone (*processus maxillaris nasalis*) is short and broad, delineating the dorsocaudal margins of the nasal openings (Figure 4A).

The premaxillary bone extends caudally, articulating with the nasal bone via the maxillary process of the nasal bone before merging with the frontal bone. Laterocaudally, it connects with the maxillary bone through the premaxillary process of the maxilla (*processus maxillaris premaxillare*). Medially, the premaxillary bone articulates with the palatine bone via the maxillary portion of the palatine (*pars maxillaris palatini*, sensu Zusi and Livezey 2006), while also joining medially with the maxillary bone (Figure 4A).

Rostrally, the maxillary bone flattens and angles downward beneath the nasal openings, thinning at its midsection and tapering toward its distal end. The distal portion of the upper jaw (*apex rostri maxillae*) curves slightly downward into a rounded tip (Figure 5A). The frontal process of the premaxillary bone (*processus frontalis premaxillares*) is flattened and angled downward, featuring numerous neurovascular foramina (*foramen neurovascularium*) on both sides. The cutting edge of the maxilla (*crista tomialis rostri maxillae*) is strongly ossified and follow the natural curvature of the bill (Figure 4A).

The ventral surface of the upper jaw is dominated by a prominent keel, originating at the premaxillary bone, where it forms a bifurcated longitudinal ridge. This keel represents a ventral projection of the fused palatine process of the premaxillary bone (*processus palatinus premaxillaris*, sensu Zusi and Livezey 2006). As it extends caudally toward the maxillary bone, the ridge gradually diminishes in prominence (Figures 3, 5). The lateral edges of the ventral surface slope upward, constituting a channel‐like structure when the jaw is closed. The palatal processes of the maxillary bone (*processus palatus maxillaris*, sensu Zusi and Livezey 2006) are well‐developed and pneumatized. The ventral portion of these processes is flattened, extending medially from the maxillary bone toward the tip of the vomer and laterally contacting the nasal bones (Figure 3B).

The maxillary portion of the palatine (*pars maxillaris palatini*, sensu Zusi and Livezey 2006) comprises a dorsoventrally flattened and relatively short palatine rostral process (*processus rostralis palatini*, sensu Zusi and Livezey 2006). This process extends rostrally to the caudal margin of the osseous nasal openings, where it is interposed between, and articulates with, both the maxillary and premaxillary bones. Just caudal to its articulation with the premaxillary bone, the palatine rostral process features a reduced bending zone (*zona flexoria palatina*, sensu Zusi and Livezey 2006). The length of the palatine rostral process, from the bending zone to the rostral margin of the choanal portion (*pars choanalis palatini*, sensu Zusi and Livezey 2006), is nearly equal to that of the palatine bone itself (Figure 3B).

The choanal portion of the palatine is continuous with the rostral process and comprises paired dorsal and ventral lamellae (*lamella dorsalis* and *lamella ventralis*, sensu Zusi and Livezey 2006). The dorsal lamellae are well‐developed and fuse to form the *crista medialis*, whereas the ventral lamellae are relatively short, project ventrally, and remain separated from each other (Figure 3B).

The lateral portion of the palatine (*pars lateralis palatini*, sensu Zusi and Livezey 2006) creates a lateral‐to‐ventrolateral expansion, which separates the dorsal and ventral lamellae. This reduced region serves as the connection between the palatine and pterygoid bones, articulating with both the caudomedial surface of the mandible and its medial process (*processus medialis mandibulae*, sensu Vanden Berge and Zweers 1993; Zusi and Livezey 2006) (Figures 3, 4).

The *pars lateralis palatini* is bounded medially by a prominent *carina ventromedialis palatini* (sensu Zusi and Livezey 2006), laterally by the *crista lateralis palatini*, and caudally by the *processus accessorius palatine*. The ventral fossa (*fossa ventralis palatini*, sensu Zusi and Livezey 2006) is elongated caudally and moderately concave. Additionally, the *angulus caudolateralis palatini* is rounded and relatively pronounced. As noted by Zusi and Livezey (2006), the ventral fossa and the lateral portion of the palatine provide the primary surface for the origin of the pterygoid muscle (*musculus pterygoideus*, sensu Vanden Berge and Zweers 1993) (Figure 3B).

The palatine is slightly separated from the pterygoid bone by the joint formed by the *articulationes pterygopalatina et intrapterygoidea* (sensu Zusi and Livezey 2006). This joint imparts flexibility to the cranial structure, playing a key role in facilitating dynamic movements associated with cranial kinesis (Zusi and Livezey 2006).

The pterygoid bone (*Os pterygoideum*) is a short and compressed bone that can be divided into three parts: the pterygoid foot (*pes pterygoidei)*, pterygoid body (*corpus pterygoidei*), and quadratic process *(processus quadraticus pterygoidei*). It is positioned between the *processus pterygoideus palatini* and the *condylus medialis quadrati*. The rostral region of the pterygoid foot, which articulates with the palatine bone, features a shallow lateral fossa adjacent to a subtly defined *facies articularis basipterygoidea*. While the *corpus pterygoidei* exhibits a rough texture across all its surfaces, the *processus quadraticus pterygoidei* is characterized by a smooth border.

The vomer is a long, thin, pointed plate that fuses with the caudal portion of the palatine. Laterally, it has an arc shape, with both caudal and cranial end sharp and its ventral portion features a thin crest along almost its entire length (Figure 3B). The jugal arch (*arcus jugalis*) is a narrow, elongated structure, dorsoventrally flattened in its rostral portion and lateromedially in its caudal portion. It is composed of an outstanding jugal process (*processus jugalis maxillare)*, the jugal bone (*Os jugale)*, and the quadratojugal bone (*Os quadratojugale*), forming a thin bar that links the quadrate (*Os quadratum*) to the upper jaw. The arch is bounded rostrally by the jugal process and articulates caudally with the quadrate through the quadratic condyle, which connects to the *cotyla quadratojugalis* (Figure 4A).

### 3.4 Os Quadratum

**Figure 6:**
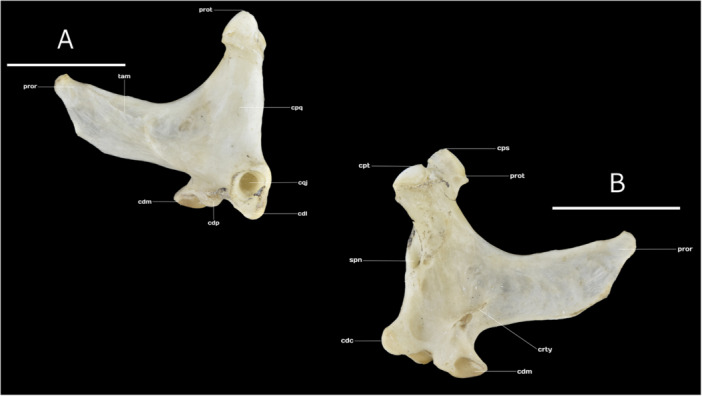
(A) Lateral external view of the Quadrate bone of Chilean Flamingo *Phoenicopterus chilensis*: **prot**, processus oticus; **tam**, tuberculum adductor mandibulae; **pror**, processus orbitalis; **cdm**, condylus medialis; **cdp**, condylus pterigoideus; **cdl**, condylus lateralis; **cqj**, condylus quadrati jugalis; **cpq**, corpus quadrati. (B) Lateral internal view of the Quadrate bone of Chilean Flamingo *P. chilensis*: **cpt**, capitulum oticum; **cps**, capitulum squamosum; **prot**, processus oticus; **pror**, processus orbitalis; **crty**, crista timpanica; **cdm**, condylus medialis; **cdc**, condylus caudalis; **spn**, sulcus pneumaticum articulare. Scale bar equal 10 mm.

The quadrate bone is a robust, fully ossified structure, characterized by three conspicuous distinct processes: the orbital (*processus orbitalis*), otic (*processus oticus*), and mandibular (*processus mandibularis*) processes. The orbital process is elongated and flattened mediolaterally, extending in a rostrodorsal into the orbit. At its base, a slight lateral tubercle (*tuberculum adductor mandibulae*) serves as the attachment site for the aponeurosis of the mandibular adductor muscle (*Musculus adductor mandibulae ossis quadrati*, sensu Vanden Berge and Zweers 1993) (Figure 6A,B).

The otic process is projected caudally and features two rounded capitula: the larger otic capitulum (*capitulum oticum*), which articulates with the caudal part of the external acoustic meatus, and the squamosal capitulum (*capitulum squamosum*), which connects with the ventral portion of the squamosal process (*processus squamosalis*, sensu Posso and Donatelli 2006) and the dorsal part of the suprameatic process (*processus suprameaticus*) of the squamosal bone. Medially, the otic process contains a small foramen (*foramen pneumaticum articulare*), located dorsally to the tympanic crest (*crista tympanica*) (Figure 6A,B).

The mandibular process comprises three condyles. The lateral condyle (*condylus lateralis)* bears the quadratojugal cotyle (*cotyla quadratojugalis)*. The medial condyle (*condylus medialis*), located medioventrally, articulates with the medial cotyle of the mandible (*cotyla medialis mandibulae*), and the caudal condyle (*condylus caudalis*) which articulates with the caudal cotyle (*cotyla caudalis*) of the lateral mandibular process (Figure 6A,B).

### 3.5 Apparatus Hyobranchialis

**Figure 7:**
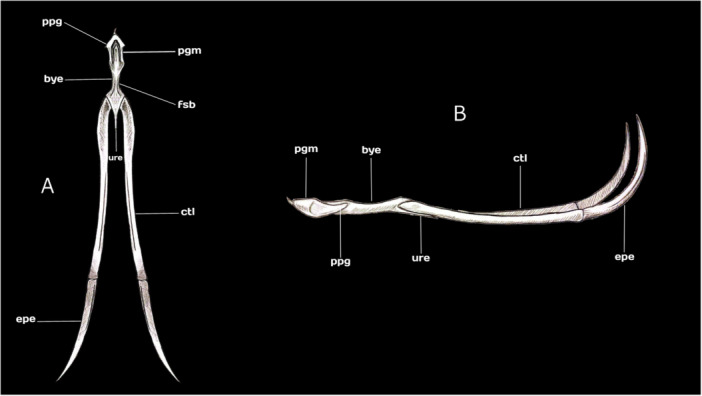
(A) Drawing overview of dorsal view of the hyoid bone of Chilean Flamingo *Phoenicopterus chilensis*: **pgm**, paraglossum; **fsb**, fossa basihyalis; **ure**, urohyale; **ctl**, ceratobranchiale; **epe**, epibranchiale; **bye**, basihyale; **ppg**, posterior process of the paraglossum. (B) Drawing overview of lateral view of the hyoid bone of Chilean Flamingo *P. chilensis*: **pgm**, paraglossum; **bye**, basihyale; **ctl**, ceratobranchiale; **epe**, epibranchiale; **ure**, urohyale; **ppg**, posterior process of the paraglossum. Scale bar equal 20 mm.

The hyoid apparatus lies between the upper jaw and the mandible, being convex cranially and concave caudally. It is large, robust, and completely embedded within the tongue, encased in soft tissue, and composed of five fully ossified and well‐developed elements: the paraglossum, basihyale, urohyale, ceratobranchiale, and epibranchiale (Figure 7A).

### 3.6 Paraglossum

The paraglossum is a solid, short bone forming the cranial portion of the hyoid. It is anchor‐shaped, with *U*‐shaped arms that project vertically and fuse cranially with the basihyale, while its posterior process is short and narrow (Figure 7A,B).

### 3.7 Basihyale

The basihyale is a short, straight, saddle‐shaped bone. Its cranial surface is fused to the paraglossum, while its caudal end is irregularly polyhedral, bearing two lateral articular facets that connect with the ceratobranchials. The proximal portion of the urohyale is centrally fused with the basihyale. The fossa basihyalis is reduced, and the lateral parahyal processes are absent (Figure 7A,B).

### 3.8 Urohyale

The urohyale is a short, thin bone projecting caudally, centrally fused with the basihyale and articulating proximally with the ceratobranchiale (Figure 7A,B).

### 3.9 Ceratobranchiale

The paired and elongated ceratobranchiale is a concave bone that articulates with the caudal part of the basihyale and with the proximal end of the urohyale, representing nearly 60% of the total length of the hyoid apparatus (Figure 7A,B).

### 3.10 Epibranchiale

The epibranchiale is a paired, thin, horn‐shaped bone that articulates caudally with the ceratobranchiale, with its sharp distal tips projecting dorsally over the ceratobranchiale (Figure 7A,B).

### 3.11 Ossa Mandibulae

The mandible of the Chilean Flamingo is divided into three distinct regions: the symphysial (*pars symphisialis)*, intermediate (*pars intermedialis*), and caudal (*pars caudalis*) parts. The symphysial part forms the rostral section, where the two halves of the mandible (*rami mandibulae*) meet and fuse at the mandibular symphysis (*symphysis mandibulae*) (Figure 5B). In the Chilean Flamingo, this symphysis is constructed solely from the ventral margins of the rami, curving downward laterorostrally at its rostral end and occupying less than one‐third of the mandible's total length. The rami are thin, tall, and pneumatized, featuring shallow lateral grooves. The cutting edge (*margo tomialis*) in this region is relatively smooth, curving downward significantly, while the tip of the mandible is blunt and rounded, containing two neurovascular openings (*foveae corpusculorum nervosa*). Ventrally, a thin keel extends along the symphysis, accompanied by a semi‐elliptical pneumatized fossa (Figure 4B).

The intermediate part accounts for approximately one‐third of the mandible's total length and is characterized by its rigidity. It extends from the downward‐curved mandibular angulus (*angulus dorsalis mandibulae*, sensu Livezey and Zusi 2006) toward the caudal part, gradually decreasing in height. The dorsal surface of this section is wide and flat, forming the paratomial plane (*planum paratomialis*, sensu Livezey and Zusi 2006), likely influenced by the contribution of the splenial bone (*Os spleniale*) (Carlos et al. 2017). The lateral surfaces are rough and contain numerous neurovascular foramina (*foramina neurovascularia*) (Figure 4B).

The caudal part is lateromedially flattened, particularly along its lateral surface (*facies lateralis*). This region includes a shallow fossa (*fossa lateralis mandibulae*) positioned anterior to a slight, elongated opening (fenestra caudalis mandibulae). The medial surface houses the deep mandibular canal fossa (*fossa aditus canalis neurovascularis*), which lies near the pseudotemporal tubercle (*tuberculum pseudotemporal*e), a small but well‐defined bony prominence that serves as the insertion site for the superficial pseudotemporal muscle (*Musculus pseudotemporalis superficialis*, sensu Vanden Berge and Zweers 1993) (Figure 4B).

The articular region of the caudal part features a narrow, deep quadratic articular fossa (*fossa articularis quadratica*), which articulates with the condyles of the quadrate bone. This fossa is divided into three sections: the medial cotyle (*cotyla medialis*), a robust and deep depression forming a hinge‐like connection with the medial condyle of the quadrate; the lateral cotyle (*cotyla lateralis*), which is elongated and shallower, accommodating the lateral condyle of the quadrate; and the caudal cotyle (*cotyla caudalis*), the shortest and shallowest of the three, containing a wide pneumatic foramen (f*oramen pneumaticum articulare*) that opens laterorostrally near the well‐developed medial process of the mandible (*processus medialis mandibulae*) (Figure 5B).

The retroarticular process (*processus retroarticularis*) extends caudally from the articular fossa as a broad, blade‐like projection. This structure features a shallow caudal fossa (*fossa caudalis*), serving as the insertion point for the depressor muscle of the mandible. The retroarticular process tapers slightly toward its tip while maintaining a broad base near the articular region. Additionally, the dorsal margin of the caudal part contains two pseudocoronoid processes (*processus pseudocoronoidei mandibulae*, sensu Donatelli 1996), which provide attachment sites for the aponeurosis of the temporal muscles (*Musculus adductor mandibulae externus* and *pars rostralis*, sensu Vanden Berge and Zweers 1993) (Figures 4, 5).

## 4 Discussion

The Chilean Flamingo possesses a unique feeding apparatus adapted for filtering food particles from shallow and intermediate water environments, a feature that underpins its specialized foraging strategies (Mascitti and Kravetz 2002; del Hoyo et al. 2024), and like other flamingo species, it exhibits a different feeding behavior, finely tuned to its diet and habitat (Delfino and Carlos 2022).

A key morphological characteristic of the Chilean Flamingo is its cranial architecture, which is notably broader than deep and distinguished by a prominent frontal region. This frontal area articulates rostrally with the craniofacial bending zone, forming a thin and narrow transverse band that functions as the sole flexion point between the skull and the upper jaw (Bock 1964; Zusi 1962). This anatomical feature permits dorsoventral rotation of the upper jaw—specifically, protraction (upward movement) and retraction (downward movement)—relative to the cranium (Zusi 1962). In flamingos, this mobility enables rapid opening and closing of the upper jaw, a function that is integral to their filter‐feeding mechanism.

In any biomechanical system, the MA indicates the relative balance between force production and movement speed (Carril et al. 2015). The low MA values observed in Chilean Flamingos in our analysis are consistent with those reported for American Flamingos by Navalón et al. (2019). Based on these results, we infer that the low MA in flamingos may be related with the reduced and shallow areas available for the insertion of the adductor muscles. Furthermore, these insertion areas are positioned relatively close to the jaw joint compared to the out‐lever, resulting in a low closing mechanical advantage. Therefore, we infer that the mandible system is not optimized for forceful biting, but rather for rapid opening/closing and suction‐pumping movements during filter feeding.

In Chilean Flamingos, as in many other birds, the development of the primary muscles that open and close the bill is often associated with the areas available for their origin and insertion (Bock 1964; Donatelli 1996; Previatto and Posso 2015). According to Navalón et al. (2019), feeding adaptation is not the main driver of bill morphological diversification in modern birds. Instead, factors such as biting mechanical advantage and body size are stronger covariates guiding the evolution of cranial structures associated with feeding. Despite this, the relationship between the cranial areas available for the development of feeding muscles and the muscles themselves may help to elucidate the specialization of foraging behaviors such as filter feeding in flamingos.

For instance, the temporal fossa in the Collared Forest Falcon (*Micrastur semitorquatus*), which preys on mammals, birds, and reptiles (Thorstrom 2000; Bierregaard et al. 2020), and the Medium Ground Finch (*Geospiza fortis*), which primarily consumes seeds (De León et al. 2014 have a large and deep surface. This structure accommodates a well‐developed adductor mandibular muscle system, enabling the Collared Forest Falcon to tear flesh and the Medium Ground Finch to crack seeds (Genbrugge et al. 2011; Da Silva et al. 2012). In contrast, the temporal fossa of the Chilean Flamingo is shallow and reduced in size. This morphology likely limits the development of large jaw muscles, favoring instead muscles adapted for rapid and finely controlled bill movements that enable the precise filter‐feeding characteristic of flamingos.

The subtemporal fossa originates the M. depressor mandibulae, and the caudal fossa of the mandible supports its insertion (Bock 1964). In the Chilean Flamingo, both fossae are reduced. This reduction may contribute to the development of slender mandibular depressor muscles, which may support the mandible and allow rapid upper jaw movement.

The tongue in flamingos is large, fleshy, and softer than in any other bird, allowing it to adjust its shape when providing the pumping force for filtration (Jenkin 1957; Zweers et al. 1995). As in most birds, the hyoid apparatus in the Chilean Flamingo is supported by cartilage and soft tissues, forming the skeletal framework of the tongue and functioning as an anchoring structure that enables its movements within the mandible (Gadow 1877; Homberger and Meyers 1989).

The paraglossum in the Chilean Flamingo is fused and anchor‐shaped, with the dorsal surface of its posterior process short and narrow, projecting along its vertical axis, unlike in most birds. This morphology appears to be associated with the reinforcement of the distal part of the tongue (Bock 1972; Homberger 1986), which likely prevents collapse when the flamingo tongue changes shape during protraction and retraction movements.

In the Chilean Flamingo, the bony basihyale exhibits interesting features: it is thin, with a smooth surface, a reduced *fossa basihyalis*, and notably, it lacks lateral parahyal processes. This suggests that the *M. hypoglossus obliquus* plays a reduced or modified role, and the tongue of flamingos does not need complex rotation or lateral narrowing. Instead, it serves as part of a semi‐rigid piston optimized for pumping water and retaining food.

The distal concave horns of the epibranchials provide areas of origin for the *M. ceratoglossus* and *M. ceratohyoideus* (Bock 1972). The synergy of these muscles, acting through the paraglossal–basihyal and cerato–basihyal joints, allows the tip of the tongue to be depressed during retraction while the ceratobranchials spread laterally, due to the limited space in the mouth floor. This coordinated movement enables the filtered food retained at the lingual base to pass into the pharynx, after which the tongue protracts to resume the filter‐feeding cycle. According to Zweers et al. (1995), the lack of space in the mouth floor is compensated for with a flexible skin of the throat that allows to bulge sharply outward when the tongue is retracted.

In the Chilean Flamingo, the ventral fossa is elongated and shallow, and the lateral portion of the palatine is also reduced in size. These relatively small structures suggest a pterygoid muscle system adapted for rapid mandibular movements. In contrast, the Rufous‐browed Peppershrike (*Cyclarhis gujanensis*) exhibits both a more developed fossa and larger pterygoid muscles, supporting greater bite forces for tearing prey (Orenstein and Barlow 1981; Previatto and Posso 2015).

The Chilean Flamingo's curved upper jaw is complemented by the mandible's distinct downward bend along its proximal half. In addition, the mandibular symphysis extends over at least 30% of the total mandible length. Simultaneously, the tubular cavity formed between the *rami* (Jenkin 1957) provides suitably space for tongue movements, thereby enhancing the efficiency of the water‐pumping system during filter feeding.

The caudal portion of the mandible, which serves as the primary attachment site for jaw musculature, exhibits a reduced pseudocoronoid processes. These diminutive processes may restrict the insertion area available for the aponeurosis of the *M. adductor mandibulae externus ventralis*, suggesting reduced mechanical demands for powerful mandibular adduction.

During their highly specialized feeding behavior, flamingos are subjected to external forces that could potentially destabilize the craniofacial hinge or the palatine–pterygoid–quadrate complex (Cracraft 1968). To counteract this, the Chilean Flamingo possesses a specialized “locking device” that protects the jaw from disarticulation (Bock 1960, 1964; Carlos et al. 2017). This mechanism involves a deep medial cotyle of the mandible, which securely encases the medial condyle, providing stability to the jaw during rapid feeding behaviors such as inverted filter feeding and stamping.

## 5 Conclusions

This study provides a detailed morphological analysis of the cranial architecture of the Chilean Flamingo and emphasizes its unique specializations for filter feeding. Our results show that the cranium is broader than it is deep, with a pronounced frontal region. This configuration allows a dorsoventral rotation of the upper jaw, facilitating rapid protraction and retraction movements. Such cranial mobility is directly associated with the flamingo's specialized filter‐feeding strategy.

The low mechanical advantage of the mandible in the Chilean Flamingo is attributed to the reduced and shallow insertion areas of the adductor muscles, which are positioned close to the jaw joint. Consequently, the jaw system is not optimized for forceful biting, but rather for rapid opening and closing movements. The reduced muscle attachment areas, such as the lateral mandibular tubercle, the pseudotemporal tubercle, the pseudocoronoid processes, and the shallow fossae for the temporal and subtemporal muscles, highlight a functional emphasis on rapid and repetitive jaw movements rather than forceful biting in its feeding apparatus.

The hyoid apparatus of the Chilean Flamingo exhibits distinct structural adaptations that reinforce the semi‐rigid, piston‐like tongue and support its role in pumping water during filter feeding. The fused, anchor‐shaped paraglossum has a reinforced distal region that prevents collapse during protraction–retraction cycles. The elongated and paired ceratobranchiales provide extensive origin sites for the *M. ceratoglossus* and *M. ceratohyoideus*. These muscles allow the tip of the tongue to be depressed during retraction. This movement enables filtered food retained at the lingual base to pass into the pharynx. Finally, the Chilean Flamingo's curved upper jaw is complemented by the mandible's downward bend along its proximal half. In addition, the tubular cavity formed between the *rami* provides a suitable space for tongue movements, enhancing the efficiency of the water‐pumping system during filter feeding.

## Author Contributions

**Oscar Aldana Ardila:** conceptualization, investigation, funding acquisition, writing – original draft, methodology, validation, visualization, writing – review and editing, formal analysis, data curation. **Caio J. Carlos:** conceptualization, investigation, validation, visualization, writing – review and editing, supervision, data curation.

## Conflicts of Interest

The authors declare no conflicts of interest.

## Supporting information


**Supporting Information Table 1:** Measurements of the skull and mechanical advantage of Chilean Flamingo *Phoenicopterus chilensis*. All measurements are in millimeters, mm.

## Data Availability

The data that supports the findings of this study are available in the supplementary material of this article.

## References

[jmor70116-bib-0001] Aldana‐Ardila, O. , and C. J. Carlos . 2021. “Feeding Ecology of the Chilean Flamingo *Phoenicopterus chilensis* (Aves: Phoenicopteridae) in a Coastal Wetland in Southern Brazil.” Journal of Natural History 55, no. 41–42: 2589–2603. 10.1080/00222933.2021.2003459.

[jmor70116-bib-0002] Baumel, J. J. , and R. J. Raikow . 1993. “Arthrologia.” In *Handbook of Avian Anatomy: Nomina Anatomica Avium*, edited by A. S. King , J. E. Breazile , H. E. Evans , and J. C. Vanden Berge .

[jmor70116-bib-0003] Baumel, J. J. , and L. M. Witmer . 1993. “Osteologia.” In *Handbook of Avian Anatomy: Nomina Anatomica Avium*, edited by A. S. King , J. E. Breazile , H. E. Evans , and J. C. Vanden Berge .

[jmor70116-bib-0004] Bierregaard, R. O. , G. M. Kirwan , and P. F. D. Boesman . 2020. “Collared Forest‐Falcon (*Micrastur semitorquatus*), Version 1.0.” In Birds of the World, edited by J. del Hoyo , A. Elliott , J. Sargatal , D. A. Christie , and E. de Juana . Cornell Lab of Ornithology.

[jmor70116-bib-0005] Bock, W. J. 1960. “Secondary Articulation of the Avian Mandible.” Auk 77, no. 1: 19–55. 10.2307/4082382.

[jmor70116-bib-0006] Bock, W. J. 1964. “Kinetics of the Avian Skull.” Journal of Morphology 114, no. 1: 1–41. 10.1002/jmor.1051140102.

[jmor70116-bib-0007] Bock, W. J. 1972. “Morphology of the Tongue Apparatus of C1R1DOPS Anna (Drepanididae).” Ibis 114: 61–78. 10.1111/j.1474-919X.1972.tb02589.x.

[jmor70116-bib-0008] Brent Gurd, D. 2006. “Filter‐Feeding Dabbling Ducks (*Anas* spp.) Can Actively Select Particles by Size.” Zoology 109, no. 2: 120–126. 10.1016/j.zool.2005.10.002. 16406531

[jmor70116-bib-0009] Burger, A. E. 1978. “Functional Anatomy of the Feeding Apparatus of Four South African Cormorants.” Zoologica Africana 13, no. 1: 81–102. 10.1080/00445096.1978.11447608.

[jmor70116-bib-0010] Carlos, C. J. , J. G. Alvarenga , and M. S. Mazzochi . 2017. “Osteology of the Feeding Apparatus of Magnificent Frigatebird *Fregata magnificens* and Brown Booby Sula leucogaster (Aves: Suliformes).” Papéis Avulsos de Zoologia 57: 265–274. 10.11606/0031-1049.2017.57.20.

[jmor70116-bib-0011] Carril, J. , F. J. Degrange , and C. P. Tambussi . 2015. “Jaw Myology and Bite Force of the Monk Parakeet (Aves, Psittaciformes).” Journal of Anatomy 227: 34–44. 10.1111/joa.12330. 26053435 PMC4475357

[jmor70116-bib-0012] Chubb, A. L. 2004. “New Nuclear Evidence for the Oldest Divergence Among Neognath Birds: The Phylogenetic Utility of ZENK (i).” Molecular Phylogenetics and Evolution 30, no. 1: 140–151. 10.1016/S1055-7903(03)00159-3. 15022765

[jmor70116-bib-0013] Cracraft, J. 1968. “The Lacrimal‐Ectethmoid Bone Complex in Birds: A Single Character Analysis.” American Midland Naturalist 80: 316–359. 10.2307/2423530.

[jmor70116-bib-0014] Cracraft, J. 1981. “Toward a Phylogenetic Classification of the Recent Birds of the World (Class Aves).” Auk 98, no. 4: 681–714. 10.1093/auk/98.4.681.

[jmor70116-bib-0015] Cracraft, J. , F. K. Barker , M. Braun , et al. 2004. “Phylogenetic Relationships Among Modern Birds (Neornithes): Toward an Avian Tree of Life.” In *Assembling the Tree of Life*, edited by J. Cracraft and M. J. Donoghue, Oxford Academic. 10.1093/oso/9780195172348.003.0028.

[jmor70116-bib-0016] Crome, F. 1985. “An Experimental Investigation of Filter‐Feeding on Zooplankton by Some Specialized Waterfowl.” Australian Journal of Zoology 33, no. 6: 849–862. 10.1071/ZO9850849.

[jmor70116-bib-0017] Dechaume‐Moncharmont, F. X. , K. Monceau , and F. Cezilly . 2011. “Sexing Birds Using Discriminant Function Analysis: A Critical Appraisal.” Auk 128, no. 1: 78–86. 10.1525/auk.2011.10129.

[jmor70116-bib-0018] Delfino, H. C. , and C. J. Carlos . 2021. “Behavioral Repertoire of a Population of Wild Chilean Flamingos *Phoenicopterus chilensis* in Southern Brazil.” Journal of Natural History 55, no. 31–32: 1957–1981. 10.1080/00222933.2021.1978574.

[jmor70116-bib-0019] Delfino, H. C. , and C. J. Carlos . 2022. “What Do We Know About Flamingo Behaviors? A Systematic Review of the Ethological Research on the Phoenicopteridae (1978–2020).” Acta Ethologica 25, no. 1: 1–14. 10.1007/s10211-021-00381-y.

[jmor70116-bib-0020] Donatelli, R. J. 1996. “The Jaw Apparatus of the Neotropical and of the Afrotropical Woodpeckers (Aves: Piciformes).” Arquivos de Zoologia 33: 1–70. 10.11606/issn.2176-7793.v33i1p1-70.

[jmor70116-bib-0021] Ericson, P. G. P. , C. L. Anderson , T. Britton , et al. 2006. “Diversification of Neoaves: Integration of Molecular Sequence Data and Fossils.” Biology Letters 2, no. 4: 543–547. 10.1098/rsbl.2006.0523. 17148284 PMC1834003

[jmor70116-bib-0022] Frias‐Soler, R. C. , A. Bauer , M. A. Grohme , et al. 2022. “Phylogeny of the Order Phoenicopteriformes and Population Genetics of the Caribbean Flamingo (*Phoenicopterus ruber*: Aves).” Zoological Journal of the Linnean Society 196, no. 4: 1485–1504. 10.1093/zoolinnean/zlac040.

[jmor70116-bib-0023] Gadow, H. 1877. “Anatomie Des *Phoenicopterus roseus* Pall. Und Seine Stellung im System.” Journal für Ornithologie 25, no. 4: 382–396.

[jmor70116-bib-0024] Gallardo, P. , and E. Rodriguez . 1992. Estudio de la alimentación de Phoenicoparrus andinus, Phoenicoparrus jamesi, y Phoenicopterus chilensis en el Salar de Surire, I Región de Tarapacá. Memoria de Título. Universidad Arturo Prat.

[jmor70116-bib-0025] Genbrugge, A. , A. Herrel , M. Boone , et al. 2011. “The Head of the Finch: The Anatomy of the Feeding System in Two Species of Finches (*Geospiza fortis* and *Padda oryzivora*): The Head of the Finch.” Journal of Anatomy 219, no. 6: 676–695. 10.1111/j.1469-7580.2011.01437.x. 21999913 PMC3237877

[jmor70116-bib-0026] Gill, F. , Donsker, D. , and Rasmussen, P. 2023. IOC World Bird List (v13.1). 10.14344/IOC.ML.13.1.

[jmor70116-bib-0027] Hackett, S. J. , R. T. Kimball , S. Reddy , et al. 2008. “A Phylogenomic Study of Birds Reveals Their Evolutionary History.” Science 320, no. 5884: 1763–1768. 10.1126/science.1157704. 18583609

[jmor70116-bib-0028] Hildebrand, M. , G. E. Goslow , and V. Hildebrand . 2006. *Análise da estrutura dos vertebrados*. Atheneu.

[jmor70116-bib-0029] Homberger, D. G. 1986. *The Lingual Apparatus of the African Grey Parrot, Psittacus erithacus Linne (Aves: Psittacidae): Description and Theoretical Mechanical Analysis*

[jmor70116-bib-0030] Homberger, D. G. , and R. A. Meyers . 1989. “Morphology of the Lingual Apparatus of the Domestic Chicken, *Gallus gallus*, With Special Attention to the Structure of the Fasciae.” American Journal of Anatomy 186, no. 3: 217–257. 2618925 10.1002/aja.1001860302

[jmor70116-bib-0031] del Hoyo, J. , P. F. D. Boesman , and E. Garcia . 2024. “Chilean Flamingo (*Phoenicopterus chilensis*), Version 1.1.” In Birds of the World, edited by J. del Hoyo , A. Elliott , J. Sargatal , D. A. Christie , and E. de Juana . Cornell Lab of Ornithology. 10.2173/bow.chifla1.01.1.

[jmor70116-bib-0032] Jarvis, E. D. , S. Mirarab , A. J. Aberer , et al. 2014. “Whole‐Genome Analyses Resolve Early Branches in the Tree of Life of Modern Birds.” Science 346, no. 6215: 1320–1331. 10.1126/science.1253451. 25504713 PMC4405904

[jmor70116-bib-0033] Jenkin, P. M. 1957. “The Filter‐Feeding and Food of Flamingoes (Phoenicopteri).” Philosophical Transactions of the Royal Society of London, Series B: Biological Sciences 240: 401–493. 10.1098/rstb.1957.0004.

[jmor70116-bib-0034] Jollie, M. T. 1957. “The Head Skeleton of the Chicken and Remarks on the Anatomy of This Region in Other Birds.” Journal of Morphology 100, no. 3: 389–436. 10.1002/jmor.1051000302.

[jmor70116-bib-0035] Klages, N. T. W. , and J. Cooper . 1992. “Bill Morphology and Diet of a Filter‐Feeding Seabird: The Broad‐Billed Prion *Pachyptila vittata* at South Atlantic Gough Island.” Journal of Zoology 227, no. 3: 385–396. 10.1111/j.1469-7998.1992.tb04401.x.

[jmor70116-bib-0036] Kouzov, S. A. , Y. I. Gubelit , A. V. Kravchuk , E. M. Koptseva , E. M. Zaynagutdinova , and V. N. Nikitina . 2021. “Seasonal Changes in the Diet of Mute Swans *Cygnus olor* in the Recently Colonized Eastern Gulf of Finland.” Wildfowl 71, no. 71: 83–107.

[jmor70116-bib-0037] De León, L. F. , J. Podos , T. Gardezi , A. Herrel , and A. P. Hendry . 2014. “Darwin's Finches and Their Diet Niches: The Sympatric Coexistence of Imperfect Generalists.” Journal of Evolutionary Biology 27, no. 6: 1093–1104. 10.1111/jeb.12383. 24750315

[jmor70116-bib-0038] Livezey, B. C. , and R. L. Zusi . 2006. “Phylogeny of Neornithes.” Bulletin of Carnegie Museum of Natural History 37: 1–544. 10.2992/0145-9058(2006)37[1:PON]2.0.CO;2.

[jmor70116-bib-0039] Manegold, A. 2006. “Two Additional Synapomorphies of Grebes Podicipedidae and Flamingos Phoenicopteridae.” Acta Ornithologica 41, no. 1: 79–82. 10.3161/068.041.0113.

[jmor70116-bib-0040] Mascitti, V. , and F. O. Kravetz . 2002. “Bill Morphology of South American Flamingos.” Condor 104, no. 1: 73–83. 10.1093/condor/104.1.73.

[jmor70116-bib-0041] Mayr, G. 2004. “Morphological Evidence for Sister Group Relationship Between Flamingos (Aves: Phoenicopteridae) and Grebes (Podicipedidae).” Zoological Journal of the Linnean Society 140, no. 2: 157–169. 10.1111/j.1096-3642.2003.00094.x.

[jmor70116-bib-0042] Montalti, D. , M. Graña Grilli , R. E. Maragliano , and G. Cassini . 2012. “The Reliability of Morphometric Discriminant Functions in Determining the Sex of Chilean Flamingos *Phoenicopterus chilensis* .” Current Zoology 58, no. 6: 851–855. 10.1093/CZOOLO/58.6.851.

[jmor70116-bib-0043] Morales‐García, N. M. , P. G. Gill , C. M. Janis , and E. J. Rayfield . 2021. “Jaw Shape and Mechanical Advantage are Indicative of Diet in Mesozoic Mammals.” Communications biology 4, no. 1: 242. 10.1038/s42003-021-01757-3. 33623117 PMC7902851

[jmor70116-bib-0044] Morgan‐Richards, M. , S. A. Trewick , and A. Bartosch‐Harlid , et al. 2008. “Bird Evolution: Testing the Metaves Clade With Six New Mitochondrial Genomes.” BMC Evolutionary Biology 8: 20. 10.1186/1471-2148-8-20. 18215323 PMC2259304

[jmor70116-bib-0045] Navalón, G. , J. A. Bright , J. Marugán‐Lobón , and E. J. Rayfield . 2019. “The Evolutionary Relationship Among Beak Shape, Mechanical Advantage, and Feeding Ecology in Modern Birds.” Evolution 73, no. 3: 422–435. 10.1111/evo.13655. 30537045

[jmor70116-bib-0046] Olson, S. L. , and A. Feduccia . 1980. *Relationships and Evolution of Flamingos (Aves, Phoenicopteridae)*.

[jmor70116-bib-0047] Orenstein, R. I. , and J. C. Barlow . 1981. *Variation in the Jaw Musculature of the Avian Family Vireonidae*. Royal Ontario Museum

[jmor70116-bib-0048] Ortega‐Jimenez, V. M. , T. Yee , P. Rohilla , B. Seleb , J. Belair , and S. Bhamla . 2025. “Flamingos Use Their L‐Shaped Beak and Morphing Feet to Induce Vortical Traps for Prey Capture.” Proceedings of the National Academy of Sciences 122, no. 21. 10.1073/pnas.2503495122. PMC12130884 40354558

[jmor70116-bib-0049] Polla, W. , V. A. Di Pasquale , M. C. Rasuk , et al. 2018. “Diet and Feeding Selectivity of the Andean Flamingo *Phoenicoparrus andinus* and Chilean Flamingo *Phoenicopterus chilensis* in Lowland Wintering Areas.” *Wildfowl* 68: 3–29. 10.1186/s40693-014-0015-1.

[jmor70116-bib-0050] Posso, S. , and R. Donatelli . 2006. “Skull and Mandible Formation in the Cuckoo (Aves, Cuculidae): Contributions to the Nomenclature in Avian Osteology and Systematics.” European Journal of Morphology 42: 163–172. 10.1080/09243860500315507. 16982472

[jmor70116-bib-0051] Previatto, D. , and S. Posso . 2015. “Jaw Musculature of *Cyclarhis gujanensis* (Aves: Vireonidae).” Brazilian Journal of Biology 75: 655–661. 10.1590/1519-6984.20113. 26421766

[jmor70116-bib-0052] Prum, R. O. , J. S. Berv , A. Dornburg , et al. 2015. “A Comprehensive Phylogeny of Birds (Aves) Using Targeted Next‐Generation DNA Sequencing.” Nature 526, no. 7574: 569–573. 10.1038/nature15697. 26444237

[jmor70116-bib-0053] Raikow, R. J. 1970. *Evolution of Diving Adaptations in the Stifftail Ducks*. University of California Press.

[jmor70116-bib-0054] Rooth, J. 1965. *The Flamingos on Bonaire (Netherlands Antilles): Habitat, Diet and Reproduction of Phoenicopterus ruber ruber Volume 41 of Natuurwetenschappelijke Studiekring voor Suriname en Nederlandse Antillen*. Selbstverl.

[jmor70116-bib-0055] Sangster, G. 2005. “A Name for the Flamingo–Grebe Clade.” Ibis 147, no. 3: 612–615. 10.1111/j.1474-919x.2005.00432.x.

[jmor70116-bib-0056] Scherer, A. L. , J. F. M. Scherer , M. V. Petry , and V. H. Valiati . 2014. “Sexual Dimorphism and Body Condition of Wintering White‐Rumped Sandpipers in Southern Brazil.” Wilson Journal of Ornithology 126, no. 3: 553–561. 10.1676/13-121.1.

[jmor70116-bib-0057] Shufeldt, R. W. 1901. “Osteology of the Flamingoes.” Annals of the Carnegie Museum 1, no. 2: 295–324.

[jmor70116-bib-0058] Sibley, C. G. , J. A. Comstock , and J. E. Ahlquist . 1990. “DNA Hybridization Evidence of Hominoid Phylogeny: A Reanalysis of the Data.” Journal of Molecular Evolution 30: 202–236. 10.1007/BF02099992. 2109085

[jmor70116-bib-0059] Da Silva, A. G. , J. B. de Ferreira , R. J. Donatelli , and A. Guzzi . 2012. “Osteologia Craniana De *Micrastur semitorquatus* Vieillot, 1817 (Falconiformes: Falconidae).” Comunicata Scientiae 3, no. 1: 64–71.

[jmor70116-bib-0060] Thorstrom, R. 2000. “The Food Habits of Sympatric Forest‐Falcons During the Breeding Season in Northeastern Guatemala.” Journal of Raptor Research 34, no. 3: 196–202.

[jmor70116-bib-0061] Tobar, C. N. , J. R. Rau , N. Fuentes , et al. 2014. “Diet of the Chilean Flamingo *Phoenicopterus chilensis* (Phoenicopteriformes: Phoenicopteridae) in a Coastal Wetland in Chiloé, Southern Chile.” Revista chilena de historia natural 87: 15. 10.1186/s40693-014-0015-1.

[jmor70116-bib-0062] Torres, C. R. , L. M. Ogawa , M. A. Gillingham , B. Ferrari , and M. van Tuinen . 2014. “A Multi‐Locus Inference of the Evolutionary Diversification of Extant Flamingos (Phoenicopteridae).” BMC Evolutionary Biology 14: 36. 10.1186/1471-2148-14-36. 24580860 PMC4016592

[jmor70116-bib-0063] Tuinenf, M. V. , D. B. Butvill , J. A. W. Kirsch , and S. B. Hedges . 2001. “Convergence and Divergence in the Evolution of Aquatic Birds.” Proceedings of the Royal Society of London. Series B: Biological Sciences 268, no. 1474: 1345–1350. 10.1098/rspb.2001.1679. PMC1088747 11429133

[jmor70116-bib-0064] Uicker, J. J. , G. R. Pennock , and J. E. Shigley . 2011. Theory of Machines and Mechanisms Oxford University Press.

[jmor70116-bib-0065] Vanden Berge, J. C. , and G. A. Zweers . 1993. “Myologia.” In *Handbook of Avian Anatomy: Nomina Anatomica Avium*, edited by A. S. King, J. E. Breazile, H. E. Evans, and J. C. Vanden Berge.

[jmor70116-bib-0066] Winkler, D. W. , S. M. Billerman , and I. J. Lovette . 2020. “Flamingos (Phoenicopteridae), Version 1.0.” In Birds of the World, edited by S. M. Billerman , B. K. Keeney , P. G. Rodewald , and T. S. Schulenberg . Cornell Lab of Ornithology. 10.2173/bow.phoeni1.01.

[jmor70116-bib-0067] Zusi, R. L. 1962. *Structural Adaptations of the Head and Neck in the Black Skimmer*. Publications of the Nuttall Ornithological Club.

[jmor70116-bib-0068] Zusi, R. L. , and B. C. Livezey . 2000. “Homology and Phylogenetic Implications of Some Enigmatic Cranial Features in Galliform and Anseriform Birds.” Annals of the Carnegie Museum 69, no. 3: 157–193.

[jmor70116-bib-0069] Zusi, R. L. , and B. C. Livezey . 2006. “Variation in the Os Palatinum and Its Structural Relation to the Palatum Osseum of Birds (Aves).” Annals of Carnegie Museum 75: 137–180. 10.2992/0097-4463(2006)75[137:VITOPA]2.0.CO;2.

[jmor70116-bib-0070] Zweers, G. , F. De Jong , and H. Berkhoudt . 1995. “Filter Feeding in Flamingos (*Phoenicopterus ruber*).” Condor 97, no. 2: 297–324. 10.2307/1369017.

